# Refractory/Resistant Cytomegalovirus Infection in Transplant Recipients: An Update

**DOI:** 10.3390/v16071085

**Published:** 2024-07-05

**Authors:** Léna Royston, Genovefa A. Papanicolaou, Dionysios Neofytos

**Affiliations:** 1 Division of Infectious Diseases, University Hospital of Geneva, 1211 Geneva, Switzerland; 2 Infectious Disease Service, Memorial Sloan Kettering Cancer Center, New York, NY 10065, USA; 3 Department of Medicine, Weill Cornell Medicine, New York, NY 10065, USA

**Keywords:** cytomegalovirus (CMV), transplant recipients, refractory infection, resistant infection, letermovir, maribavir

## Abstract

Despite the significant progress made, CMV infection is one of the most frequent infectious complications in transplant recipients. CMV infections that become refractory or resistant (R/R) to the available antiviral drugs constitute a clinical challenge and are associated with increased morbidity and mortality. Novel anti-CMV therapies have been recently developed and introduced in clinical practice, which may improve the treatment of these infections. In this review, we summarize the treatment options for R/R CMV infections in adult hematopoietic cell transplant and solid organ transplant recipients, with a special focus on newly available antiviral agents with anti-CMV activity, including maribavir and letermovir.

## 1. Introduction

Cytomegalovirus (CMV) infection constitutes a major burden for transplant recipients, associated with significant morbidity and mortality. As a member of the *Herpesviridae* family, CMV establishes a lifelong latency in hematopoietic progenitor cells, with frequent subclinical reactivation events and subsequent infection of numerous cell types in varied organs. With an estimated seroprevalence from 50 to 90% and high rates of reactivation after transplantation, CMV infection is believed to constitute the most frequent opportunistic infection in both hematopoietic cell (HCT) and solid organ transplant (SOT) recipients [1]. Despite decades of research and significant advancements in CMV infection management, many challenges remain. In particular, refractory or resistant (R/R) infections to the available anti-CMV drugs are frequently encountered, emphasizing the need for new and better-tolerated agents. Nevertheless, new anti-CMV therapies have recently been developed and introduced in clinical practice, which may subsequently radically change the landscape of CMV infection in the near future. In this review, we aim to summarize the treatment options for R/R CMV infections in adult HCT and SOT recipients, with a special focus on newly available antiviral agents with anti-CMV activity, including maribavir and letermovir.

## 2. Current Definitions and Management of CMV Infections in Transplant Recipients

For consistency in clinical trial design and to standardize clinical guidelines, clear definitions of CMV infections have been proposed by dedicated study groups [2,3]. As such, CMV infection is defined as the detection of viral proteins or nucleic acids or as virus isolation from any fluid or specimen. Recurrent infection refers to the reemergence of CMV infection in a patient who had no evidence of CMV detection during a period of 4 weeks of active viral surveillance. CMV disease is defined as the evidence of additional signs or symptoms attributable to CMV replication, including any end-organ disease (pneumonia, gastrointestinal disease, hepatitis, and other). Depending on the detection of CMV on tissue biopsy or not, CMV disease may be further defined as proven or probable, respectively. CMV syndrome, which is used only in SOT recipients, is defined as CMV detection in blood and ≥2 signs or symptoms including, but not limited to, fever, malaise/fatigue, leukopenia/neutropenia, atypical lymphocytosis, thrombocytopenia, or liver enzyme elevation. Refractory CMV infection refers to the persistence of infection despite administration of appropriate antiviral treatment, mostly presenting as an increase (of at least 1 log10) or the persistence of CMV DNAemia in blood with less than 1 log10 reduction after 2 weeks of well-conducted antiviral treatment [4]. Finally, resistant CMV infection is defined as the detection of a viral mutation conferring a reduced sensitivity to at least one anti-CMV agent [4].

## 3. Underlying Mechanisms and Epidemiology of Refractory/Resistant CMV Infections

Refractory or resistant CMV infections are difficult to manage and are associated with increased morbidity and mortality in transplant recipients [5,6,7]. Prolonged antiviral therapy, especially at lower doses with subtherapeutic blood levels, has been associated with the emergence of resistance. Additional risk factors for CMV resistance emergence in transplant recipients include the level of immunosuppression, rejection, the donor/recipient CMV serostatus, high CMV viral loads, or recurrent infections [8,9,10]. In SOT recipients, the risk of resistance also depends on the type of immunosuppressive regimen and to the transplanted organ, with lung transplantation being at higher risk [8]. For HCT recipients, additional risk factors include the presence of graft-versus-host disease (GvHD) and T-cell depleted grafts [10]. Resistance to ganciclovir and valganciclovir mostly arise in the UL97 gene, coding for the viral kinases needed for drug activation, and less frequently in UL54, the viral polymerase itself [11]. As CMV polymerase constitutes the ultimate target of all these drugs, mutations in UL54 may confer cross-resistance to ganciclovir, valganciclovir, foscarnet, and cidofovir. The degree of resistance induced depends on the codon mutated, ranging from high-level resistance (as for A594V mutation), low-level resistance (e.g., C592G mutation), insignificant resistance (e.g., A591V mutation), or even mutations considered as baseline polymorphisms with no significant resistance induced (e.g., Q449K mutation) [11,12]. In SOT recipients, ganciclovir resistance is estimated to occur in 5–22% of all patients, with lung transplant recipients being at higher risk [8,13,14]. In HCT recipients, reported rates of resistance range from 1.7 to 14.5% of patients [5,15]. The frequency of resistance to other antiviral drugs, such as foscarnet and cidofovir, is less well documented.

In one-third to one-half of patients with refractory CMV infection, resistance-conferring mutations are, however, not detected [7,16]. The cause of these suboptimal responses to treatment could be multifactorial, including severe immunosuppression or suboptimal drug exposure.

The epidemiology of refractory CMV infections with or without resistance mutations is difficult to estimate, due to the different diagnostic criteria that have been used thus far. In general, the incidence of R/R infection is considered to be higher in SOT than in HCT recipients, due to the almost routine administration of (val)ganciclovir as anti-CMV prophylaxis and hence more frequent and longer exposure in SOT recipients to anti-CMV active agents used for treatment of CMV. However, in a recent Spanish systematic review of 19 studies, R/R CMV infection rates were estimated to be in the range of 3–10% in SOT recipients, and 11.3–50% in HCT recipients [17]. Wide ranges of definitions used in clinical practice and trials may explain this gap and prevent a clear view of R/R burden. It should also be noted that most of those data come from the pre-letermovir era. The introduction of universal anti-CMV prophylaxis with letermovir during the first 3–6 months after an allogeneic HCT has significantly decreased the frequency of clinically significant (cs) CMV infection post-transplant [18,19,20,21,22]. The significantly lower rates of csCMV infection observed in allogeneic HCT recipients in the current era have led to lower rates of R/R CMV infection in this patient population [23]. However, even if the exact incidence of these infections remains ill-defined, R/R CMV infections are surely encountered and, when they occur, they constitute a major challenge for the clinical management of these patients.

## 4. Clinical Management of R/R CMV Infections

Until recently, only CMV DNA polymerase (UL54) inhibitors were available, and those drugs still constitute the first line for CMV treatment. Ganciclovir and its oral prodrug valganciclovir are guanine analogs that first need to be phosphorylated by a viral kinase, UL97, to further inhibit CMV DNA replication by acting as chain terminators. Foscarnet and cidofovir are pyrophosphate and cytidine monophosphate analogs, respectively. Whereas foscarnet directly blocks the pyrophosphate binding site on DNA polymerase, cidofovir needs to be activated by cell kinases to further act as a chain terminator. Although effective in reducing CMV replication, the use of these drugs is largely limited by their inherent toxicities, including myelotoxicity, nephrotoxicity, electrolyte abnormalities, and mucosal ulceration [24,25]. In addition, and as already mentioned above, the emergence of resistance remains a relatively frequent complication. Until the recent development of new anti-CMV drugs, the clinical management of R/R infections was complex, as only few second-line and toxic options were available. An important step in the management of R/R infection is the identification of specific resistance-conferring mutations by genotypic sequencing to inform treatment decisions [12]. In the case of CMV-resistant mutations in UL97 (or some codons in UL54) conferring resistance to ganciclovir only, the switch to foscarnet could be considered. In the presence of UL54 mutants, resistance to all available options could emerge, leading to desperate treatment options, such as combining (val)ganciclovir with foscarnet with additive toxicities and poor clinical outcomes [26]. The reduction in immunosuppression, when feasible, may further contribute to improved clinical outcomes, although by itself not sufficient enough to clear the infection. Recent data suggest that CMV-specific T cell immunity could potentially inform decisions about duration of make it possible to stratify patient risk for discontinuation of CMV prophylaxis or preemptive treatment and need for secondary prophylaxis [27,28,29]. Well-designed studies are needed to validate the safety and potential utility of this approach.

## 5. New CMV Antivirals

In recent years, novel targets in the CMV replication cycle have emerged along with anti-viral drugs, targeting other steps than only DNA replication, through polymerase inhibition.

As for other herpesviruses, DNA synthesis of CMV is processed by the rolling cycle mechanism, which leads to DNA concatemers. The viral terminase complex, constituted by UL56, UL89, and UL51, further cleaves those concatemers to package them into the preformed capsids. Letermovir is a specific inhibitor of the UL56 subunit of the terminase complex, preventing the cleavage of concatemeric DNA into genomic units, encapsidation, and thus the formation of CMV mature virions [30,31]. Another key viral protein targeted by maribavir is UL97, which is a serine/threonine kinase that phosphorylates multiple host and viral proteins [32]. Most importantly, this protein is involved in viral nuclear egress through the phosphorylation of nuclear lamina components, resulting in the accumulation of immature virions in the nucleus [33]. However, other steps of the infection are also inhibited, such as encapsidation and DNA replication.

## 6. Maribavir: A New Approved Option for R/R CMV Infections

With a multimodal antiviral effect, maribavir, a benzimidazole riboside, was initially developed more than 20 years ago, but was only recently approved for the treatment of R/R CMV infections [34]. Competitively inhibiting UL97, which is required to activate ganciclovir, this drug has shown an activity in vitro against CMV but also EBV, but with no effect on other herpesviruses and an antagonistic effect on ganciclovir action [35]. In animal studies, the oral bioavailability of maribavir was excellent and the safety profile was favorable [36]. The development of this drug in clinical studies was then hampered by mixed results coming from dosing modifications. While early trials using prophylactic maribavir at lower doses failed to show efficacy in HCT and SOT recipients, a phase 2 dose-escalating study in HCT recipients reported a good efficacy, but a high rate of side effects, especially dysgeusia, at higher doses. Following the results of a prospective, randomized, double-blind, dose-ranging, phase 2 clinical trial of maribavir as treatment for R/R CMV infections in SOT and HCT recipients, a multi-center, randomized, phase 3 clinical trial was recently completed, in which maribavir was compared to investigator-assigned therapy (ganciclovir, valganciclovir, foscarnet, cidofovir, or a combination) as treatment of R/R CMV infection in high-risk transplant recipients [37]. This study demonstrated the superiority of maribavir to the comparator, with CMV DNAemia clearance achieved in 55.7% at week 8 in the maribavir group versus 23.9% in the investigator-assigned therapy group (*p* < 0.001), and a maintained effect at week 16 (*p* = 0.01) [16]. Apart from dysgeusia, which was frequent (37.2%) but temporary and fully reversible in the maribavir arm, the maribavir safety profile was significantly better, especially regarding nephrotoxicity (8.5% versus 21.3% for foscarnet) and neutropenia (9.4% versus 33.9% for ganciclovir/valganciclovir). This trial led to the approval of maribavir by the FDA and other relevant medical agencies for R/R CMV infections in both HCT and SOT recipients [38].

## 7. Maribavir Strengths and Limitations/Resistance

With a different mode of action, maribavir has the advantage of being an interesting alternative in case of multiple resistances to first- or second-line treatments. Its good bioavailability and oral administration render it usable in outpatient settings, thus avoiding hospitalization and associated costs. In addition, its safety profile, compared to all other available treatment options, makes it a desirable treatment option. Of note, the taste disturbances, observed in almost a third of patients treated with this agent, may still impact the patients’ quality of life, particularly in patients with upper gastrointestinal (GIT) GvHD. It is also important to be aware of the fact that maribavir does not cross the brain–blood barrier and thus cannot be used for the treatment of CMV encephalitis or retinitis. Finally, it should not be used in combination with (val)ganciclovir due to antagonist interaction between these two agents. Preclinical and clinical studies have also suggested that maribavir may have an intermediate genetic barrier to resistance [13]. Although assessed in the pivotal clinical trial by Avery et al., a more detailed analysis of that study reported that 26% (60/234 participants) of patients with R/R CMV who received up to 8 weeks of maribavir therapy developed genotypic evidence of maribavir resistance [39]. Emergence of resistance was found in 48% of non-responders and 86% of those who initially cleared the virus but rebounded while still on maribavir. In total, four different mutations on UL97 were retrieved, conferring different levels of resistance. Interestingly, two of these mutations conferred a dual resistance to maribavir but also to ganciclovir (with, respectively, 2.3-fold and 6-fold increased ganciclovir EC_50_).

## 8. Letermovir

In preclinical studies, letermovir demonstrated a high in vitro activity against CMV, including clinical isolates resistant to other drugs, with very low EC50 values (1.6–5.1 nM) and low toxicity, but no activity against other herpesviruses [40,41]. In phase IIa/b trials, the pharmacokinetic parameters, efficacy, and safety of letermovir were tested in SOT and HCT recipients, demonstrating a good tolerance and efficacy in suppressing CMV replication [23,42]. This led to a phase 3 trial, showing a reduction in the proportion of patients with clinically significant CMV infections from 61% to 38% at week 24 in CMV seropositive (R+) allogeneic HCT recipients receiving letermovir 480 mg once daily for 14 weeks post-HCT compared to controls receiving placebo [18]. This primary endpoint included patients without detectable CMV DNA at randomization, and patients who discontinued the trial for any reason before week 24 after transplantation or who had missing data at week 24 were imputed as having a primary end-point event. In addition, a survival benefit was reported in patients receiving letermovir, without any difference in the frequency or severity of adverse events between cases and controls. Based on this pivotal study, letermovir 480 mg once daily (or 240 mg if co-administered with cyclosporine) has been since then recommended as primary prophylaxis in CMV R+ HCT recipients for 14 weeks post-HCT. However, reports emerged on the increased incidence of late CMV infections after letermovir discontinuation [43,44,45]. For instance, our group reported a 23% incidence of csCMV infection during the first 100 days after letermovir discontinuation, which predominately occurred in haploidentical HCT recipients [45]. In a study by Zamora and colleagues, letermovir was shown to be associated with a delay in CMV-specific immune reconstitution at 3 months post-HCT, compared to preemptive therapy [46]. By measuring T-cell polyfunctional responses, the authors could show that CMV peak DNAemia and sustained viral shedding while on letermovir were associated with stronger polyfunctional T-cell responses, and subsequently decreased risks of late CMV infections. This led to a recent clinical trial, aiming at prolonging letermovir prophylaxis duration. This study demonstrated a similar benefit on reducing the incidence of clinically significant CMV infection when letermovir was administered for the first 200 versus 100 days post-allogeneic HCT, although an effect on mortality was not observed and rates of clinically significant CMV infection at 48 weeks post HCT were similar between the two groups [19]. In SOT recipients, a large phase 3 trial tested the efficacy of letermovir compared to valganciclovir prophylaxis in 601 CMV D+/R- kidney transplant recipients until week 28 after transplantation [47]. Letermovir was shown to be non-inferior in preventing CMV disease and exhibited lower rates of adverse events, especially leukopenia (11.3 vs. 37%), leading to the FDA approval for this indication in June 2023 [48].

In parallel, many real-world clinical trials have confirmed the efficacy of letermovir prophylaxis in transplant recipient cohorts worldwide, and also suggested a benefit of letermovir prophylaxis on other post-transplant outcomes, as non-CMV infections or immune reconstitution [20,49]. Small reports or case series have also reported on letermovir as preemptive treatment or treatment for R/R CMV, either alone or in combination with other agents [50]. A recent review of studies on the use of letermovir for the treatment of R/R infections estimated that sustained virological control was documented in 76% of those infections, whereas treatment failure was reported in 24% of cases [50]. One study including 27 SOT and 21 HCT recipients suggested that virologic outcome was better when letermovir was initiated at CMV viral loads <1000 IU/mL [51]. More recently, data have emerged on the off-label use of letermovir as secondary prophylaxis in patients at risk for CMV recurrence [52,53]. However, in the absence of large clinical studies, more data on the potential utility of letermovir in combination with other modalities in the management of R/R CMV infection or as secondary prophylaxis in high-risk transplant patients are urgently needed.

## 9. Letermovir Strengths and Limitations/Resistance

Letermovir is available in both oral and IV formulations. Its oral bioavailability is excellent, even in patients with severe GIT GvHD [54]. Data on therapeutic drug monitoring suggest that orally administered letermovir, even in patients with severe GIT GvHD, is adequately absorbed [54]. Nevertheless, large variability has been reported in blood concentrations of this agent. In addition, letermovir is well-tolerated, even in patients with hematologic or renal impairments. Pharmacological interactions with cyclosporine are well-reported and require dose adjustments. Furthermore, interactions with other drugs, such as posaconazole or corticosteroids, have also been suggested in real-world studies and should be monitored [54]. Due to the unique mechanism of action and molecular target of letermovir, there is no cross-resistance between letermovir and other anti-CMV drugs if these agents are used concurrently. While letermovir is currently approved only for prophylaxis, it has been used in clinical practice for the treatment of resistant CMV infections with limited or no other options. The emergence of resistance to letermovir in the setting of active CMV replication constitutes a potential disadvantage of this drug as a treatment option. Resistance mutations to letermovir emerge mostly in the UL56 gene, although more rarely also in the UL51 and UL89 genes, and do not confer cross-resistance to other anti-CMV drugs. Compared to other drugs, in vitro selection of resistant CMV strains by letermovir is rapidly achieved, with UL56 mutants arising after only five passages, suggesting a low genetic barrier to resistance [55]. However, in vivo, the rate of resistance emergence during letermovir prophylaxis is very low. For instance, in the pivotal phase 3 study of Marty et al., a retrospective analysis of the patients with CMV breakthrough infections showed a very low rate (3/50 genotyped strains) of letermovir-resistant CMV variants detected [56]. Letermovir resistance was also exceedingly low when prophylaxis was given for 200 days in HCT or kidney transplant recipients [19,47]. In contrast, letermovir resistance has been reported when used for treatment. Carefully designed clinical studies investigating the safety, dosage, and rate of emergence of drug resistance are required to delineate the optimal use of letermovir as adjunct therapy in the treatment of HCMV infections that are refractory or resistant to currently available drugs.

## 10. Other Therapeutical Options and Perspectives

Brincidofovir (BCV, HDP-CDV) is comprised of a lipid moiety, 3-hexadecyloxy-1-propanol (HDP), conjugated to the phosphonate of cidofovir (CDV). BCV retains the broad-spectrum activity of CDV against dsDNA viruses, while lipid conjugation counters two major limitations of CDV, namely nephrotoxicity and the lack of oral bioavailability [57,58,59]. Phase 1 studies established safety and tolerability and a phase 2 study showed good efficacy for CMV prophylaxis post-HCT [60,61]. SUPPRESS was a phase 3 randomized, double-blind, placebo-controlled trial, which evaluated oral brincidofovir CMV prophylaxis in CMV-seropositive HCT recipients for 14 weeks, with a primary endpoint of clinically significant infection at 24 weeks [62]. While at the end of 14 weeks the brincidofovir arm had significantly less CMV infections, at week 24 CMV infections were similar between the brincidofovir (51.2%) and placebo (52.3%) groups [62]. The brincidofovir arm had higher rates of gastrointestinal toxicities, GvHD, leading to higher empiric use of corticosteroids. As a result, further development of oral brincidofovir for prophylaxis or treatment was halted due to those dose-limiting toxicities. In 2021, oral brincidofovir was approved by the FDA for treatment of smallpox as single-dose administration [63]. An IV formulation of brincidofovir is currently under investigation. In a phase 2 dose escalating study of IV brincidofovir for the treatment of adenovirus infection (including 70% HCT recipients), no gastrointestinal toxicity or nephrotoxicity were observed [64]. Further development of IV brincidofovir for the treatment of CMV is planned.

Anti-CMV monoclonal antibodies may represent complementary options to prevent or treat CMV infections in association with other antiviral agents. Purified from pooled plasma from donors with high anti-CMV IgG titers, there are currently two types of CMV immunoglobulins that are available, namely Cytogam^®^ in the US and Cytotect^®^ in Europe. Although pre-clinical studies showed a good efficacy in neutralizing CMV infective particles and preventing CMV infection in animal models, routine administration of CMV immunoglobulin as adjunct therapy for CMV infections is not recommended [65,66], although it could occasionally be considered in cases of severe CMV pneumonitis.

Regarding vaccines against CMV, although they could constitute an interesting tool to prevent R/R infections, there is still no effective vaccine available, as well as no adequate correlates of protective immunity despite decades of research [67].

Finally, adoptive CMV T-cell therapy constitutes an appealing option, as T-cell immunity is essential in CMV control. The adoptive transfer of HLA-matched or partially matched CMV-specific T-cells from a CMV seropositive donor, expanded ex vivo to restore T-cell immunity, has been explored in HCT since the early 1990s [68,69]. However, clinical evidence still relies to date only on small phase 1 or phase 2 studies [50]. Some of these reports showed a benefit in challenging situations of CMV R/R infections [50]. In HCT recipients, the infusion of CMV-specific T-cells originating from the donor or third-party donors have shown an efficacy of about 70% in treating R/R CMV infections with good safety outcomes [70]. In addition to treating CMV, a potential added advantage of this approach is the durability of both complete and partial responses contributed by the CMV specific T-cells of the donor or recipient origin [71]. Regarding SOT recipients, only few patients have been treated with such therapies, with even less evidence regarding efficacy and safety [50]. Access remains an important issue, as well as the cost and time constraints, that make this therapy even more challenging to use. A larger phase 3 study and more data on optimized protocols and cell preparation may be needed to include antiviral T-cell therapy in clinical guidelines.

## 11. Conclusions

Important advances have been made in recent years regarding CMV infection management in transplant recipients. Resistant/refractory CMV infection, however, still constitutes a challenging situation for physicians, requiring second-line treatments that are based on less evidence and clinical experience. While the conventional CMV antivirals, namely ganciclovir, foscarnet, and cidofovir, target the DNA polymerase, maribavir and letermovir have different molecular targets with limited or no cross-resistance to DNA polymerase inhibitors. Furthermore, these newer antivirals are orally bioavailable, safe, and well-tolerated, thus expanding our armamentarium of anti-CMV antivirals. Due to the fact that the molecular targets for maribavir and letermovir are downstream of DNA replication, these drugs are more vulnerable to the development of resistance due to continued limited CMV replication when used for the treatment of CMV infections. Maribavir is now approved for the treatment of R/R CMV infections in HCT and SOT recipients, based on the good efficacy and safety results compared to standard therapy in a large pivotal phase 3 study [16]. Maribavir should thus be considered as the first option for CMV infections that are refractory or resistant to ganciclovir or foscarnet. However, it should be kept in mind that the level of resistance emergence is probably not negligeable, especially in patients with sustained CMV viral loads while on treatment, and that this drug does not pass the blood–brain barrier. Regarding letermovir, very few data have been published so far regarding its efficacy in treating R/R infections. Its lower efficacy in durability of virologic responses, particularly when starting treatment with high CMV viral loads, may constitute a drawback, although this remains to be proven in larger studies. Available data on letermovir for secondary prophylaxis are promising and merit further investigation. Finally, despite the few data available, adoptive T-cell therapies could constitute an interesting option in the future for particularly difficult to treat R/R CMV and other viral infections.

## Abbreviations

CMV: cytomegalovirus; R/R: refractory or resistant; GvHD: graft-versus-host disease; HCT: hematopoietic cell transplantation; SOT: solid organ transplantation.
